# Structural studies of WDR5 in complex with MBD3C WIN motif reveal a unique binding mode

**DOI:** 10.1016/j.jbc.2024.107468

**Published:** 2024-06-12

**Authors:** Yang Yang, Li Xu, Shuting Zhang, Liangrui Yao, Yuqing Ding, Wenwen Li, Xuemin Chen

**Affiliations:** 1 School of Life Sciences, Anhui University, Hefei, Anhui, China; 2 Institute of Biotechnology and Health, Beijing Academy of Science and Technology, Beijing, China

**Keywords:** WDR5, MBD3C, WIN motif, crystal structure, unique binding mode

## Abstract

The nucleosome remodeling and deacetylase (NuRD) complex plays a pivotal role in chromatin regulation and transcriptional repression. In mice, methyl-CpG binding domain 3 isoform C (MBD3C) interacts specifically with the histone H3 binding protein WD repeat-containing protein 5 (WDR5) and forms the WDR5-MBD3C/Norde complex. Despite the functional significance of this interaction on embryonic stem cell gene regulation, the molecular mechanism underlying MBD3C recognition by WDR5 remains elusive. Here, we determined the crystal structure of WDR5 in complex with the peptide (residues 40–51) derived from the MBD3C protein at a resolution of 1.9 Å. Structural analysis revealed that MBD3C utilizes a unique binding mode to interact with WDR5, wherein MBD3C Arg43 and Phe47 are involved in recognizing the WDR5-interacting (WIN) site and Tyr191-related B site on the small surface of WDR5, respectively. Notably, the binding induces a ∼91° rotation of WDR5 Tyr191, generating the hydrophobic B site. Furthermore, mutation experiments combined with isothermal titration calorimetry (ITC) assays confirmed the importance of both Arg43 and Phe47 in mediating WDR5 binding affinity. By determining structures of various peptides bound to WDR5, we demonstrated that the WDR5 WIN site and B site can be concurrently recognized by WIN motif peptides containing ′′Arg-Cies/Ser-Arg-Val-Phe′′ consensus sequence. Overall, this study reveals the structural basis for the formation of the WDR5-MBD3C subcomplex and provides new insights into the recognition mode of WDR5 for the WIN motif. Moreover, these findings shed light on structural-based designs of WDR5-targeted anti-cancer small molecule inhibitors or peptide-mimic drugs.

WDR5, a well-established core scaffolding protein found in histone methyltransferase complexes MLL1-4 and SET1A/B (1, 2), was initially characterized as an epigenetic histone “writer” to recognize histone H3 lysine 4 (H3K4) and is essential for H3K4 di- and tri-methylation (H3K4me2 and H3K4me3) to activate gene transcription (1, 3, 4, 5, 6). Moreover, the interaction between WDR5 and the H3 tail could be regulated by the methylation of H3 arginine 2 (H3R2) (7, 8). It is also present in the nonspecific lethal (NSL) complex of histone acetyltransferase, where it is responsible for depositing acetylation on histone H4 lysine 16 (H4K16) (9). Two separate and non-overlapping sites located on opposite surfaces of WDR5 mediate most of its interactions with binding partners: an arginine-bearing cavity located on the surface known as the WDR5-interacting (WIN) site, and a shallow cleft on the opposite side referred to as the WDR5-binding (WBM) site (10, 11). Over the last decade, more than 100 crystal structures of WDR5 in complex with cofactors or small molecule inhibitors have been deposited into the Protein Data Bank (PDB). Interestingly, two recent studies have demonstrated that WDR5 could also interact with long noncoding RNAs (lncRNAs) through the WIN site (12, 13). In addition to its role in H3K4 methylation, WDR5 is involved in various other physiological processes, including the promotion of osteoblast and chondrocyte differentiation (14), maintenance of embryonic stem (ES) cells pluripotency and self-renewal (1, 15), and the facilitation of chromosome congression and proper spindle assembly during mitosis (16, 17). However, increasing evidence has shown that WDR5 is upregulated in a range of human cancers and plays a crucial role in cancer cell proliferation (18, 19), including prostate cancer (20, 21), breast cancer (22), leukemia (23), pancreatic cancer, and neuroblastoma (24, 25). Its overexpression is frequently associated with poor prognoses (22, 23). Thus, WDR5 emerges as a promising candidate for pharmacological inhibition in malignancy (10, 26, 27). In recent years, several small-molecule inhibitors have been developed to target WDR5 WIN and WBM sites to either obstruct the interaction or promote the degradation of WDR5 (27, 28, 29). For example, Yu *et al.* (2021) demonstrated that the specific WDR5 degrader MS67 effectively reduced WDR5 levels in cells and suppressed tumor growth in patient-derived mouse models (30). In addition, peptide-mimetic drugs have been employed to target arginine-binding pockets or hydrophobic cavities located on the opposite side (31, 32).

MBD3, a core component of the NuRD corepressor complex (33), plays a specific role in regulating the pluripotency of embryonic stem cells (34, 35). In contrast to MBD2, which can bind to methylated CpG sequences, MBD3 lacks the capacity to bind DNA with methylation (36). However, two groups revealed that MBD3C is a 5-methylcytosine (5 mC) or 5-hydroxymethylcytosine (5hmC) binder through structural investigation and electrophoretic mobility shift assays (EMSAs) (37, 38). Additionally, previous studies have shown that MBD3 was enriched at the bivalent genes, indicating a potential role for MBD3 in regulating gene expression through function with Polycomb or MLL/SET1 complexes (38, 39). ES cells lacking Mbd3 are viable and maintain normal self-renewal properties but exhibit defects in lineage commitment potential (35). In mouse ES cells, the MBD3 protein has three distinct isoforms, MBD3A, MBD3B, and MBD3C, which are primarily distinguished by variations in their amino termini (40). MBD3A is characterized by the existence of a methyl-binding domain, which is truncated in MBD3B and completely absent in MBD3C. However, MBD3C features a unique N-terminal region consisting of 50 amino acids, which includes a WIN motif and is exclusively present in ES cells, diminishing as the cells undergo differentiation (40). Recently, MBD3C was identified as a novel WDR5-binding protein by Ly-Sha *et al.* (2017) through biochemical assays (40). This discovery suggests that the complex may have a role in maintaining the stem-like state, thus establishing a connection between WDR5 and the NuRD complex. Notably, the result was in line with findings from prior studies conducted by Bode *et al.* (2016) (41). Comparing the sequence of the WIN motif of MBD3C with previously determined ones (2, 9, 42, 43, 44), we found that the residues from the +2 to +4 position (when the conserved arginine is denoted as position 0) exhibited significant differences (Fig. S1*A*), which raises the question of how WDR5 specifically recognizes the MBD3C WIN motif.

In this study, we used X-ray crystallography to determine the structure of WDR5 complexed with the MBD3C WIN motif (residues 40–51). The structural analysis revealed that WDR5 interacts with MBD3C in a unique manner. In comparison to previously reported WIN motif-containing peptides (3, 9, 42, 43, 44, 45, 46), MBD3C demonstrates binding not only to the WIN site but also to the B site, which is formed by an alternative orientation of WDR5 Tyr191. Furthermore, our binding assays have shown the importance of recognizing both the WDR5 WIN site and B site by MBD3C. To further elucidate the recognition of WDR5 by MBD3C, we have determined additional five high-resolution crystal structures, including WDR5+MBD3C_40-51_ (F47A), WDR5+SET1B (residues 1745–1754), WDR5+SET1B_1745-1754_ (E1750R/G1751V), WDR5+MBD3C_40-51_ (R45E/V46G), and WDR5+MBD3C_40-51_ (C44S/R45E/V46G). Based on these findings, we propose that our structural investigation would provide valuable insights for the development of small molecule inhibitors and peptide-based drugs.

## Results

### Overall structure of WDR5 in complex with the MBD3C peptide

**Figure 1:**
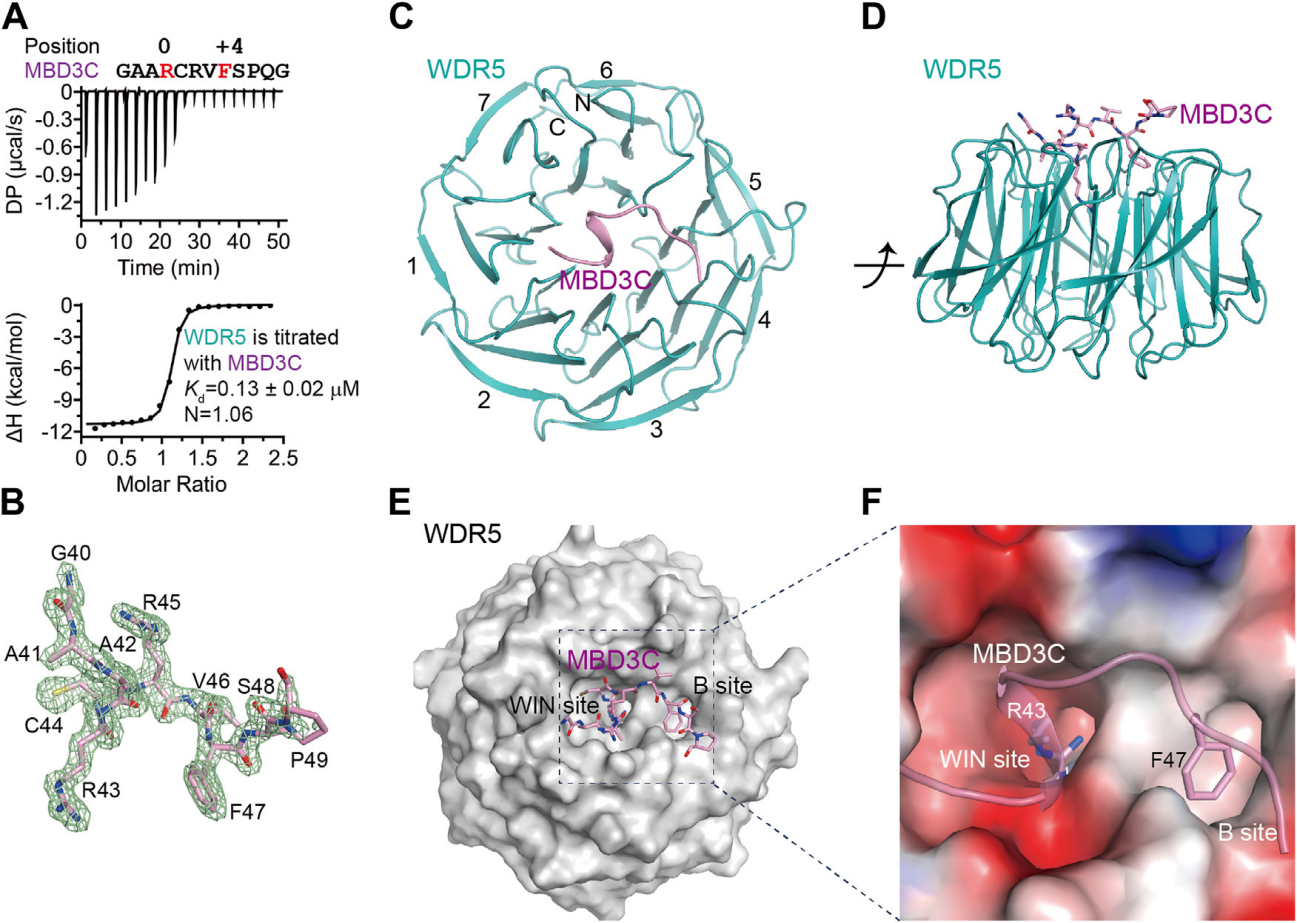
**WDR5 binds MBD3C using two totally different pockets.***A*, ITC measurement of the interaction between WDR5 and MBD3C peptide. *B*, the 2Fo-Fc omit map of MBD3C peptide bound to the WDR5 protein contoured at 1.5 σ level. *C* and *D*, overview of the WDR5-MBD3C complex. WDR5 and MBD3C are colored in *cyan* and *violet*, respectively. MBD3C is shown as a *cartoon and sticks* in (*C* and *D*), respectively. *E*, surface representation of the MBD3C-binding surface of WDR5 in a view same to *C*. The MBD3C peptide is depicted in stick representation. The WIN site and B site are labeled. *F*, close-up view of electrostatic surface representation of the MBD3C-binding surface of WDR5. MBD3C peptide is shown as a cartoon with 60% transparency. The Arg43 and Phe47 of MBD3C are shown as *sticks*.

A previous study suggested that the amino acid sequence 40 to 51 (_40_GAARCRVFSPQG_51_) of MBD3C plays a role in mediating WDR5 binding in a coimmunoprecipitation (Co-IP) experiment (40). To validate their findings and determine the dissociation constant (*K*_d_) between WDR5 and MBD3C_40-51_ more accurately, we performed ITC experiments using a synthetic MBD3C peptide spanning 40 to 51 to titrate the truncated WDR5 construct (residues 24–334). The ITC experiment data revealed a direct binding between the peptide and WDR5 with a stoichiometry of approximately 1:1, and the *K*_d_ value was 0.13 μM (Fig. 1*A*).

To gain insight into the recognition of MBD3C_40-51_ by WDR5, we determined the crystal structure of truncated WDR5 in a complex with the MBD3C_40-51_ peptide. The complex crystallized in space group *C*2_1_, and the structure was resolved at 1.9 Å by molecular replacement using the structure of apo-form WDR5 (PDB code: 2H14) as a search model (3). The structure was refined with Rwork and Rfree values of 18.22% and 20.91% (Table 1), respectively.

**Table 1:** Data collection and refinement statistics

Protein	WDR5-MBD3C	WDR5-MBD3C	WDR5-MBD3C R45E/V46G
		F47A	
PDB code	8WXQ	8WXR	8WXT
Data Collection			
Wavelength(Å)	0.979	0.979	0.979
Space group	*C*2_1_	*P*2_1_	*C*222_1_
Cell parameters			
a, b, c (Å)	116.349, 47.42, 129.474	64.697, 47.028, 103.05	78.442, 98.831, 80.178
α, β, γ (°)	90, 113.18, 90	90, 107.469, 90	90, 90, 90
Resolution∗ (Å)^a^	40.00–1.90 (1.93–1.90)	50.00–2.10 (2.14–2.10)	50.00–1.83 (1.86–1.83)
Rmerge (%)	12.3 (51.7)	4.5 (18.1)	6.7 (32.4)
CC1/2	(0.528)	(0.907)	(0.929)
I/σI	12.76 (1.86)	16.52 (5.62)	32.53 (5.97)
Completeness (%)	98.3 (85.8)	90.3 (82.1)	97.7 (99.8)
Redundancy	6.3 (5.0)	2.3 (1.6)	7.3 (5.7)
Refinement			
No. reflections used/free	50,398/5086	31,827/3103	27,227/1324
Resolution (Å)	39.67–1.90	12.65–2.08	6.87–1.83
*R*_work_^b^/*R*_free_^c^ (%)	18.22/20.91	15.27/19.86	15.09/17.91
R.m.s.deviations			
Bonds lengths (Å)	0.005	0.008	0.006
Bond angles (˚)	0.868	1.022	0.938
*B*-factors (Å^2^)			
Protein	30.80	19.21	18.21
Water	35.69	25.08	31.66
No. atoms			
Protein	4816	4799	2424
Water	390	383	340
Ramachandran plot			
Favored/allowed/outlier (%)	96.42/3.58/0	96.42/3.58/0	95.79/4.21/0


	WDR5-SET1B	WDR5-SET1B E1750R/G1751V	WDR5-MBD3C C44S/R45E/V46G
PDB code	8WXV	8WXX	8WXU
Data Collection			
Wavelength(Å)	0.979	0.979	0.979
Space group	*P*1	*C*2_1_	*C*222_1_
Cell parameters			
a, b, c (Å)	46.904, 61.499, 64.774	115.801, 47.244, 129.675	78.694, 98.917, 79.791
α, β, γ (°)	110.338, 91.273, 112.388	90, 112.965, 90	90, 90, 90
Resolution∗ (Å)	50.00–2.40 (2.44–2.40)	50.00–2.10 (2.14–2.10)	50.00–2.40 (2.44–2.40)
Rmerge (%)	9.7 (29.2)	9.5 (47.4)	11.5 (51.3)
CC1/2	(0.920)	(0.824)	(0.852)
I/σI	9.47 (2.7)	13.35 (2.19)	11.15 (2.81)
Completeness (%)	98.0 (97.0)	99.2 (97.0)	98.7 (99.7)
Redundancy	3.4 (3.2)	3.8 (2.6)	3.9 (3.5)
Refinement			
No. reflections used/free	23,652/2406	37,928/3654	11,979/1272
Resolution (Å)	33.54–2.40	27.21–2.10	39.90–2.37
*R*_work_^b^/*R*_free_^c^ (%)	16.76/20.53	17.37/21.16	17.76/23.34
R.m.s.deviations			
Bonds lengths (Å)	0.004	0.007	0.004
Bond angles (˚)	0.815	1.127	0.803
*B*-factors (Å^2^)			
Protein	35.73	29.65	35.47
Water	34.79	33.84	35.73
No. atoms			
Protein	4724	4831	2430
Water	220	391	98
Ramachandran plot			
Favored/allowed/outlier (%)	96.53/3.47/0	96.75/3.25/0	95.15/4.85/0

a Values in parentheses are for highest-resolution shell.

b Rwork = ∑hkl| |Fobs|−|Fcalc | |/∑hkl|Fobs|, where Fobs and Fcalc are the observed and calculated structure-factor amplitudes, respectively.

c Rfree is calculated the same as Rwork with 5% reflections, which were selected randomly from the refinement process.

There were two WDR5-MBD3C complexes per crystallographic asymmetric unit with a root-mean-square deviation (RMSD) of 0.140 Å between 277 pairs of Cα atoms. Thus, we performed a structural analysis based on one of the two WDR5 molecules (chain A) and the corresponding MBD3C peptide. Nine residues (Gly40-Pro49) located at the N-terminus of the MBD3C peptide were successfully fitted into the electron density (Figs. 1*B*, S1, *B* and *C*), while the last two residues (Qln50-Gly51) at the C-terminus could not be constructed due to weak electron density. The residues Val31-Cys334 of WDR5 are visible and consist of seven WD40 repeats that form a seven-bladed β-propeller structure (Fig. 1*C*). The analysis of the MBD3C_40-51_ binding mode revealed that the peptide adopts an elongated conformation, featuring a short 3_10_ α-helix at the N-terminus, positioned across the central channel on the smaller surface of the β-propeller disc (Fig. 1*D*). Consistent with our hypothesis, MBD3C is roughly located in the same position as previously proposed for the binding of H3, SET1A/B, and MLL1-4 peptides (3, 4, 5, 6, 43, 44). Analyzed by the electrostatic potential surface, we found that MBD3C occupied two distinct pockets on the smaller surface of WDR5. Specifically, MBD3C was observed to occupy an arginine-binding pocket (WIN site) with its basic Arg43, and a hydrophobic pocket (B site) with its Phe47 (Fig. 1, *E* and *F*).

### WDR5 binds MBD3C_40-51_ through two distinct binding pockets

**Figure 2:**
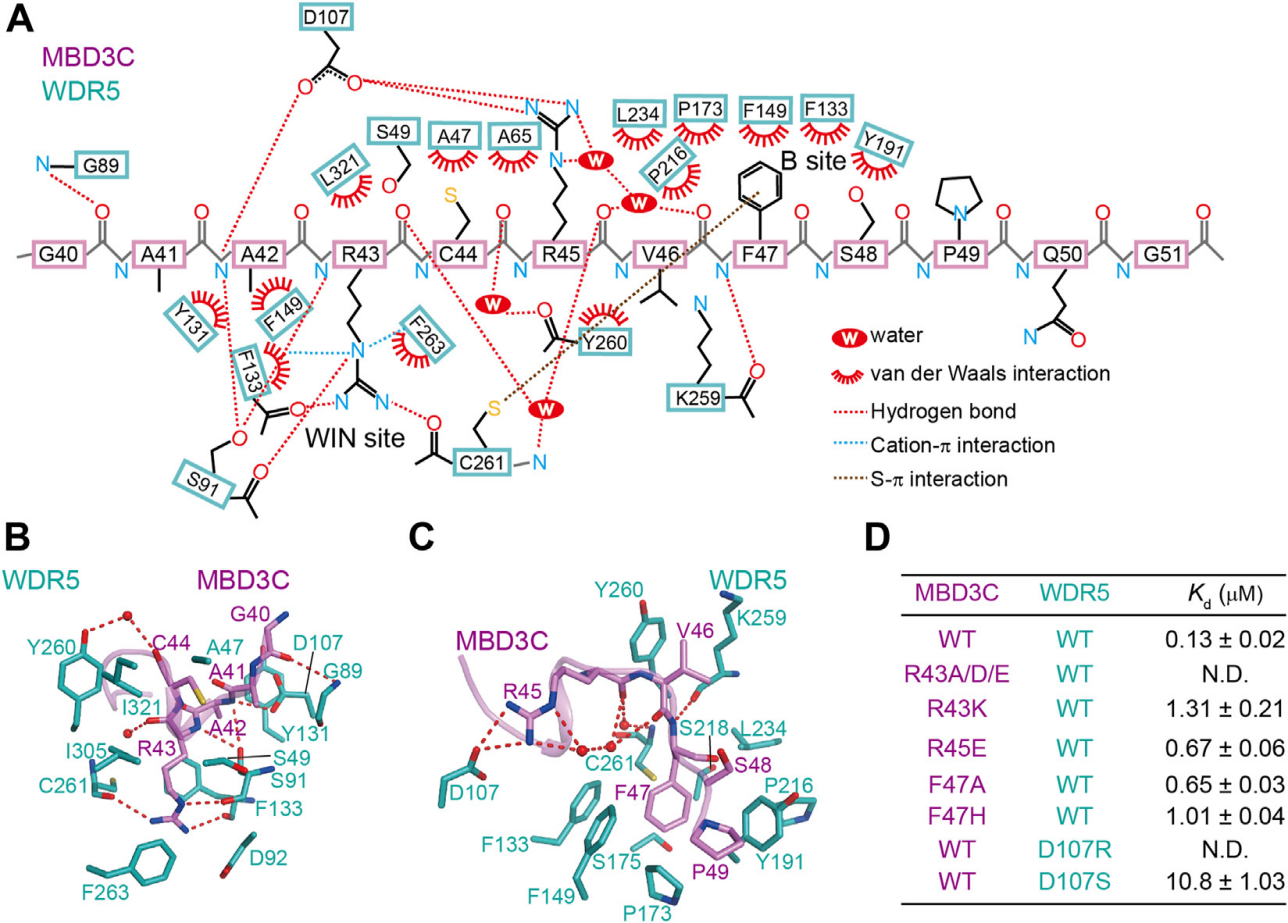
**Interaction details between WDR5 and MBD3C.***A*, schematic representation of interactions between WDR5 and MBD3C. The color codes for the WDR5 and MBD3C peptides are defined as described in Figure 1*C*. *B*, close-up view of the binding interface between WDR5 and MBD3C residues spanning 40 to 44. Hydrogen bonds are indicated by *red dashed lines*. The red spheres represent water. *C*, close-up view of the binding interface between WDR5 and MBD3C residues spanning 45 to 49. *D*, dissociation constants (*K*d) for the indicated interactions obtained by ITC assays.

The MBD3C peptide is anchored into its two binding pockets at the WDR5 binding cleft by a hydrogen-bond network and van der Waals contacts (Fig. 2*A*). To provide a more concise explanation of WDR5-MBD3C interactions, the detailed description has been divided into two parts: residues 40 to 44 and 45 to 49 of MBD3C, corresponding to the WIN site and B site, respectively.

For the WDR5-MBD3C_40–44_ interactions (Fig. 2, *A* and *B*), Gly40 in the MBD3C peptide interacts with WDR5 through its backbone carbonyl oxygen, forming a hydrogen bond with the main chain of Gly89. The backbone amide group of MBD3C Ala42 generates hydrogen bonds with the carboxyl group of WDR5 Asp107 and the hydroxyl group of WDR5 Ser91. In addition, the methyl group of Ala42 engages in hydrophobic interactions with the side chains of WDR5 Tyr131, Phe133, and Phe149. MBD3C Arg43 directly inserts into the WIN site, located within the central channel of the WDR5 β-propeller. Furthermore, the guanidinium group is sandwiched between the aromatic side chains of Phe133 and Phe263 within this cavity, leading to the formation of cation-π interactions (47). The guanidinium group of Arg43 is further stabilized through hydrogen-bonding interactions with the backbone carbonyl oxygen of Phe133, Cys261, and Ser91. In addition, the side chain of MBD3C Cys44 also interacts with WDR5 Ala47, Ser49, Ala65, and Leu321 *via* van der Waals contacts.

For the WDR5-MBD3C_45–49_ interactions (Fig. 2, *A* and *C*), Arg45 in the peptide interacts with WDR5 *via* its basic side chain, forming salt bridges with the carboxyl group of Asp107. In addition, van der Waals contacts occur between the side chain of Val46 of the MBD3C peptide and the aliphatic parts of WDR5 Lys259 and Tyr260. The backbone amide group of MBD3C Phe47 in the peptide forms a hydrogen bond with the carbonyl oxygen of WDR5 Lys259. Moreover, the aromatic ring of Phe47 stretched into the WDR5 hydrophobic B site, forming by Phe133, Phe149, Pro173, Ser175, Tyr191, Pro216, Ser218, Leu234, and Cys261. It is noteworthy that the sulfhydryl group of Cys261 also generates S-π interaction with the aromatic ring of Phe47 (48). In addition, the side chains of MBD3C Ser48 and Pro49 form interactions with WDR5 Tyr191 *via* van der Waals contacts. In addition to the aforementioned direct interactions between WDR5 and MBD3C, water-mediated hydrogen-bonding networks were also observed at the interface of WDR5-MBD3C.

To validate the importance of the aforementioned residues in the interactions between MBD3C and WDR5, we conducted mutations on Arg43, Arg45, and Phe47 residues within MBD3C and the corresponding residues within WDR5. Using ITC, we demonstrated that no binding was observed for the MBD3C peptide containing the R43A, R43D, and R43E mutations (Figs. 2*D*, S2, and Table 2). In addition, MBD3C carrying R43K mutation weakens the interaction with WDR5 by ∼9.1-fold (Figs. 2*D*, S2, and Table 2). This suggests that the presence of Arg43 in MBD3C is essential for its binding to WDR5. No interaction was observed upon substitution of Asp107 of WDR5 with arginine (Figs. 2*D*, S3, S4, and Table 2), in which the electrostatic and hydrogen-bonding interactions were both eliminated. Furthermore, the replacement of the Asp107 with serine substantially decreased the binding affinity by ∼83.1-fold (Figs. 2*D*, S3, S4, and Table 2). The Arg-Asp specific interaction was also observed in a previously determined WDR5-KANSL1 complex, thereby contributing to the high binding affinity of KANSL1 for WDR5 (9). Moreover, substituting Arg45 with glutamic acid, which introduces electrostatic repulsion to WDR5 Asp107, results in WDR5 binding with a *K*_d_ value of 0.67 μM, 4.2-fold weaker than that of the wild-type MBD3C (Figs. 2*D*, S2, and Table 2). This demonstrates the importance of MBD3C Arg45 in its binding to WDR5. In comparison to the wild-type MBD3C, the dissociation constants were approximately 4-fold and 6.8-fold higher when Phe47 was mutated to alanine and histidine (Figs. 2*D*, S2, and Table 2), respectively. Nevertheless, these results suggest the importance of MBD3C Phe47 recognition of the WDR5 B site.

**Table 2:** ITC results in 200 mM NaCl solution

Protein	Peptide	△H (kcal/mol)	−T△S (kcal/mol)	N	*K*_d_ (μM)
WDR5	MBD3C	−11.4 ± 0.13	2.13	1.06	0.13 ± 0.02
WDR5	MBD3C^R43A^				N.D.
WDR5	MBD3C^R43D^				N.D.
WDR5	MBD3C^R43E^				N.D.
WDR5	MBD3C^R43K^	−15.0 ± 0.52	7.09	1.17	1.31 ± 0.21
WDR5	MBD3C^R45E^	−7.49 ± 0.08	−0.80	1.25	0.67 ± 0.06
WDR5	MBD3C^F47A^	−7.32 ± 0.05	−0.98	1.34	0.65 ± 0.03
WDR5	MBD3C^F47H^	−7.80 ± 0.04	−0.24	0.99	1.01 ± 0.04
WDR5^D107R^	MBD3C				N.D.
WDR5^D107S^	MBD3C	−5.40 ± 0.17	−1.26	1.34	10.8 ± 1.03

*K*_d_, N, ΔH, −TΔS stand for dissociation constant, binding stoichiometry, binding enthalpy and entropy, respectively. Each *K*_d_ value is presented as a fitted value ± error.

N.D, not detectable binding.

Each experiment was performed in triplicate. Dissociation constants (*K*_d_s) were from a minimum of three experiments (mean ± SD).

Taken together, the combined findings from structural analysis and ITC data demonstrated that MBD3C Arg43 was essential for WDR5 recognition, while MBD3C Phe47 enhanced its binding affinity.

### Unique binding mode of WDR5-MBD3C

Structural analysis of various WIN motif-containing peptides bound to WDR5, we found that the WIN motif of SET1B (_1745_GCARSEGFYT_1754_) is the only one featuring an ′′Arg-X-X-X-Phe′′ pattern, as MBD3C does (43, 44). To better understand the binding mechanism of WDR5-MBD3C, we set out to determine the crystal structure of the WIN motif of SET1B (residues 1745–1754) in complex with WDR5 at 2.40 Å resolution (Table 1). The structure of the WDR5-SET1B complex closely resembles that of previously reported models (PDB code: 3UVO, 4ES0) (43, 44).

**Figure 3:**
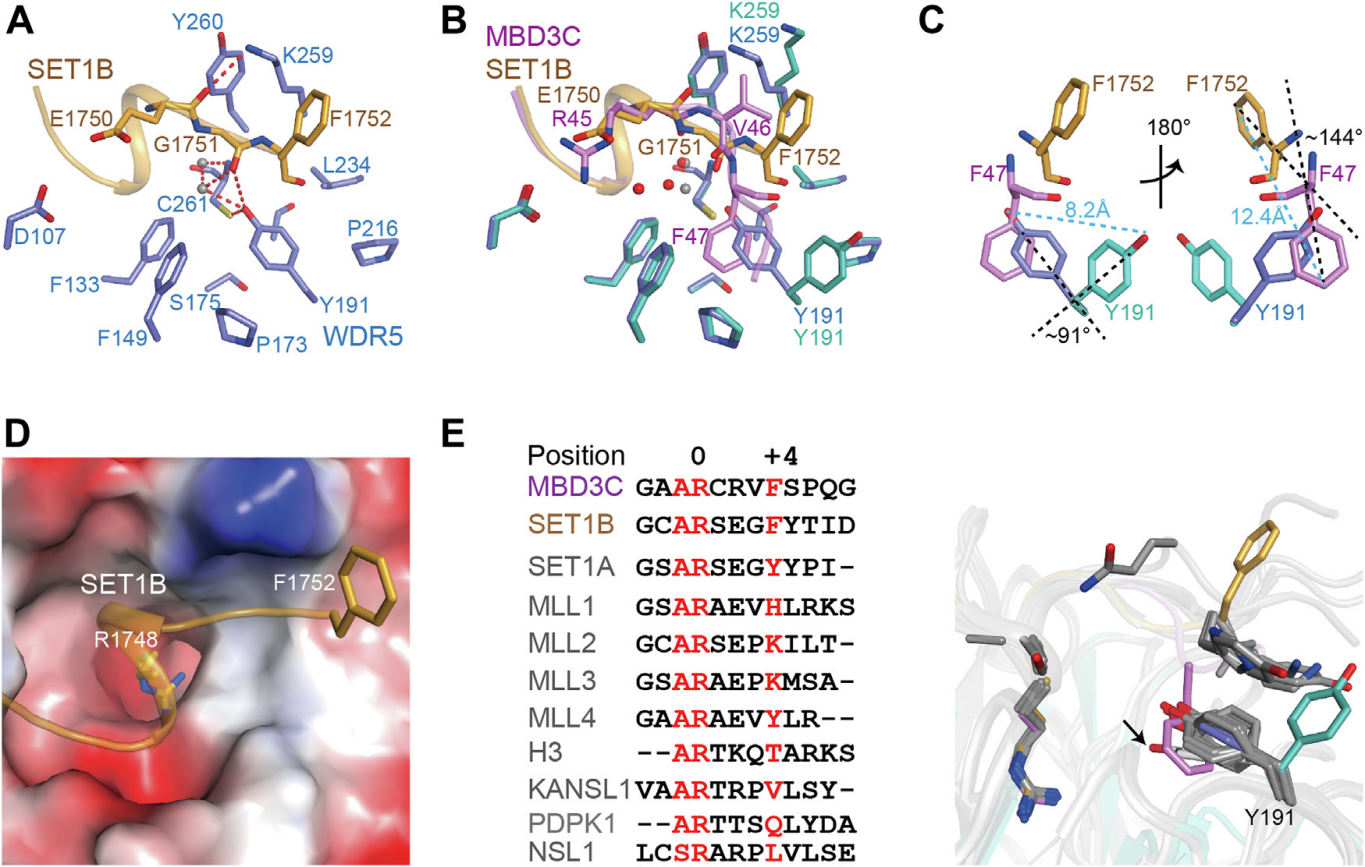
**Unique binding mode of WDR5-MBD3C.***A*, close-up view of the binding surface between WDR5 and SET1B residues spanning 1745 to 1754. WDR5 and SET1B are colored in *slate* and *bright orange*, respectively. The color code for hydrogen bonds is defined as described in Figure 1*A*. *Gray spheres* represent water. *B*, superposition of WDR5-MBD3C and WDR5-SET1B. The RMSD is 0.157 Å among 281 Cα atoms. The *gray spheres* represent waters in the WDR5-SET1B complex, while the *red spheres* represent waters in WDR5-MBD3C complex. *C*, compared to SET1B bound WDR5 Tyr191, MBD3C bound WDR5 Tyr191 rotates about 91° along its Cβ-Cζ axis and drives an 8.2 Å movement of the hydroxyl group in the sidechain of Tyr191. Compared to WDR5-bound SET1B Phe1752, the MBD3C Phe47 rotates about 144° along its Cβ-Cζ axis, resulting in a 12.4 Å movement of phenylalanine Cζ atom. *D*, electrostatic surface representation of the SET1B bound surface of WDR5. SET1B Arg1748 and Phe1752 are labeled with sticks. *E*, sequence alignment of WIN motif-containing peptides and structure superposition of WDR5-peptide complexes. The conformation of Tyr191 in different complexes was highlighted. Y191 of apo WDR5 is shown in *gray stick* and labeled with a *black arrow*.

Structural alignment revealed that SET1B interacts with WDR5 in a manner distinct from MBD3C, which is mainly located at the C-terminal region of peptides (Fig. 3, *A* and *B*). In the structure of WDR5-SET1B, acidic residue Glu1750 is unable to form salt bridges with WDR5 Asp107, unlike MBD3C Arg45. Instead of forming hydrogen bonds with water molecules, the Glu1750 carbonyl is flipped in the opposite direction and generates a hydrogen bond with the ε-NH_2_ group of WDR5 Lys259 (Fig. 3*A*). In comparison to water molecules in WDR5-MBD3C, two water molecules adjacent to WDR5 Cys261 in WDR5-SET1B move and generate a hydrogen bond network with WDR5 Tyr191, which is further stabilized by the hydrogen-bonding interaction of WDR5 Tyr191 with Cys261. The most significant structural difference lies in the ∼91° rotation of WDR5 Tyr191 from the MBD3C-bound state to SET1B-bound state (Fig. 3*C*). Consequently, the hydrophobic B site formed by the rotation of Tyr191 in WDR5 is closed in WDR5-SET1B (Fig. 3*D*), and the side chain of SET1B Phe1752 is oriented towards the solution, thus making minimal contribution to the interaction with WDR5.

In the apo structure of WDR5 (PDB code: 2H14, Fig. S5), the WDR5 Tyr191 is also headed to Cys261, leading to the closure of the B site similar to the conformation observed in SET1B-bound WDR5. The MBD3C-binding site on WDR5 has previously been shown to be bound by a WIN motif-containing histone H3 (PDB code: 2H9M), MLL1 (PDB code: 3EG6), MLL2 (PDB code: 3UVK), MLL3 (PDB code: 3UVL), MLL4 (PDB code: 3UVM), SET1A (PDB code: 3UVN), SET1B (PDB code: 3UVO), NSL1 (PDB code: 4CY3), KANSL1 (PDB code: 4CY1) and PDPK1 (PDB code: 6WJQ) (6, 9, 42, 43, 49). To validate the structural specificity of MBD3C in its binding to WDR5, the structures of various WDR5-peptide complexes were superimposed. The results suggest that, with the exception of MBD3C, all other peptides exhibit a similar WDR5 Tyr191 binding mode as SET1B (Fig. 3*E*). This indicates that the binding mode of MBD3C is distinct from the previously determined WDR5-WIN motif structures. Overall, our data highlight the unique WDR5 binding mode of MBD3C.

### Molecular basis of the unique WDR5 binding mode of MBD3C

Based on the above structural analysis, it is probable that the regulation of WDR5 Tyr191 in the MBD3C-binding state is affected by water molecules in close proximity to WDR5 Cys261, MBD3C Phe47, and the residues connecting Arg43 and Phe47. Several structures of WDR5 in complex with a series of mutated MBD3C peptides were determined to comprehend the underlying molecular basis of the WDR5 B site recognition.

**Figure 4:**
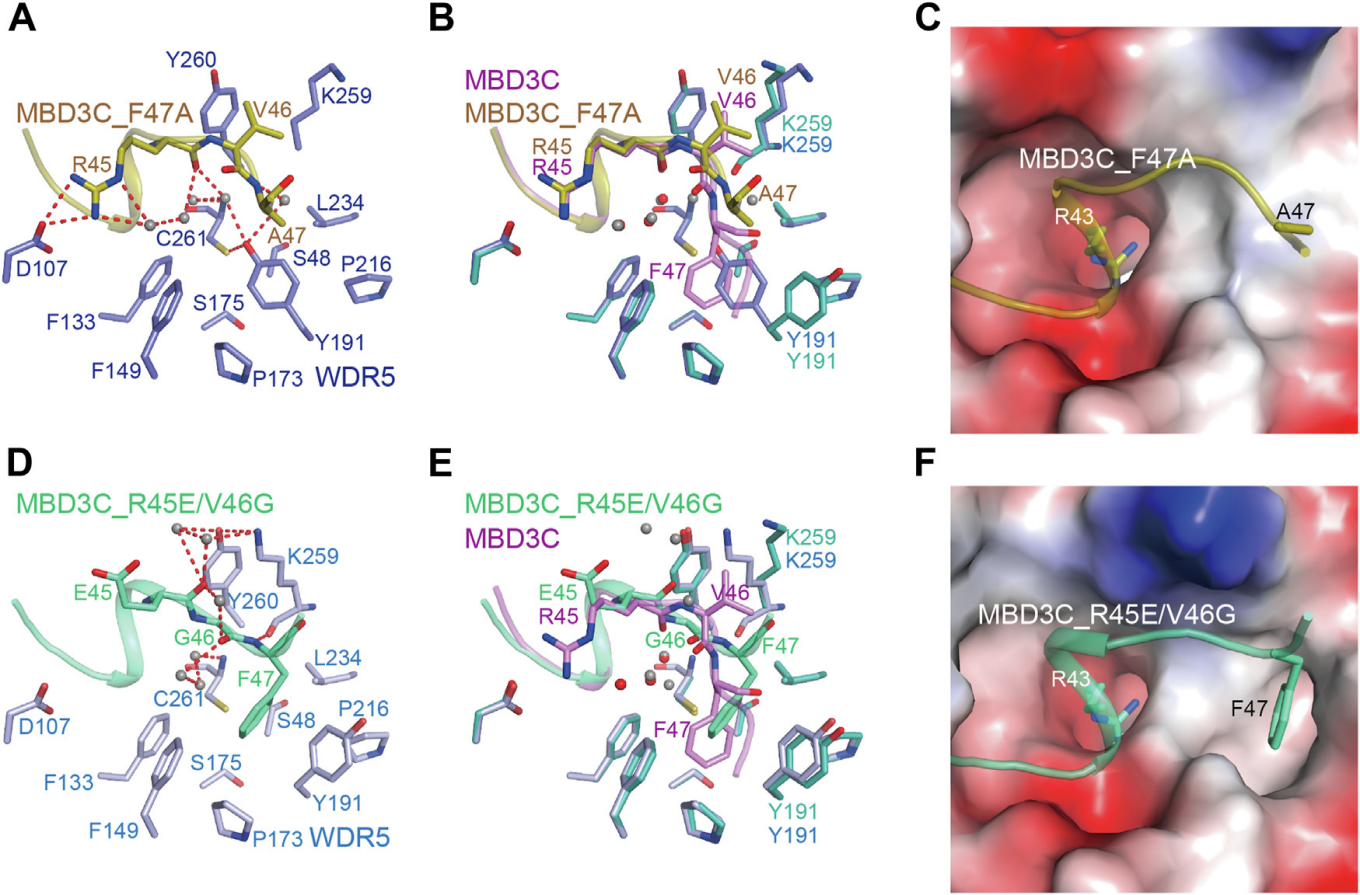
**Interaction details between WDR5 and MBD3C mutated variants.***A*, details of the binding surface between WDR5 and MBD3C_F47A variant peptide. WDR5 and MBD3C_F47A are colored in *slate* and *deep-olive*, respectively. The color code for hydrogen bonds is defined as described in Figure 1*A*. *B*, superposition of WDR5-MBD3C and WDR5-MBD3C_F47A. The RMSD is 0.112 Å among 278 Cα atoms. *C*, electrostatic surface representation of the MBD3C F47A bound surface of WDR5. Arg43 and Ala47 of MBD3C_F47A variant are shown with *sticks*. *D*, details of the binding surface between WDR5 and MBD3C_R45E/V46G variant peptide. WDR5 and MBD3C_R45E/V46G are colored in *light blue* and *green-cyan*, respectively. *E*, superposition of WDR5-MBD3C and WDR5-MBD3C_R45E/V46G. The RMSD is 0.175 Å among 258 Cα atoms. *F*, electrostatic surface representation of the MBD3C_R45E/V46G bound surface of WDR5. Arg43 and Phe47 of MBD3C_R45E/V46G are shown with *sticks*.

First, we solved the structure of WDR5-MBD3C_40-51_ (F47A) at 2.10 Å resolution (Table 1). In line with our exception, Arg45 and Val46 of MBD3C_40-51_ (F47A) interact with WDR5 in a manner similar to that of the wild-type MBD3C (Fig. 4, *A*–*C*). However, the WDR5 Tyr191 in WDR5-MBD3C_40-51_ (F47A) adopts a closed conformation and generates a hydrogen-bond interaction with Cys261. In addition, a new water molecule is introduced into the vicinity of WDR5 Cys261, leading to the formation of a hydrogen bond network that closely resembles the one observed in the WDR5-SET1B structure. Thus, these data indicated that MBD3C Phe47 is crucial for the opening and recognition of the WDR5 B site.

Next, we determined the structure of WDR5-MBD3C_40-51_ (R45E/V46G) at 1.83 Å resolution (Table 1). Glu45 and Gly46 of MBD3C_40-51_ (R45E/V46G) exhibit a distinct interaction with WDR5 compared to the wild-type MBD3C, resembling the interaction pattern observed in SET1B-WDR5 complex (Figs. 3*A* and 4, *D*–*F*). Although a new water molecule is introduced into the vicinity of WDR5 Cys261, it does not have the ability to generate a hydrogen bond with WDR5 Tyr191. In this complex structure, the WDR5 B site remains accessible on the surface of MBD3C_40-51_ (R45E/V46G) bound to WDR5. Nevertheless, the Phe47 residue in the mutated MBD3C exhibits a 2.2 Å outward displacement from the WDR5 B site, leading to a notable reduction in its interaction area with this pocket.

To provide additional evidence of the conformational change of Phe47, we then determined the structure of WDR5-MBD3C_40-51_ (C44S/R45E/V46G) (Table 1). The structure closely resembled that of WDR5-MBD3C_40-51_ (R45E/V46G) (Fig. 6, *A*–*C*). Taken together, these data collectively indicated that both MBD3C Phe47 and the residues connecting MBD3C Arg43 and Phe47 are crucial for its interaction with the WDR5 B site.

### Modified SET1B recognizes the WDR5 B site in the same way as MBD3C

Although SET1B contains a similar ′′Arg-X-X-X-Phe ′′ motif, our data has confirmed that SET1B does not induce the formation of the WDR5 Tyr191-related B site. Based on the structural analysis provided earlier, we hypothesized that amino acid substitution within the SET1B peptide sequence will alter its binding mode with WDR5. To test this hypothesis, we crystallized the WDR5-SET1B_1745-1754_ (E1750R/G1751V) complex.

**Figure 5:**
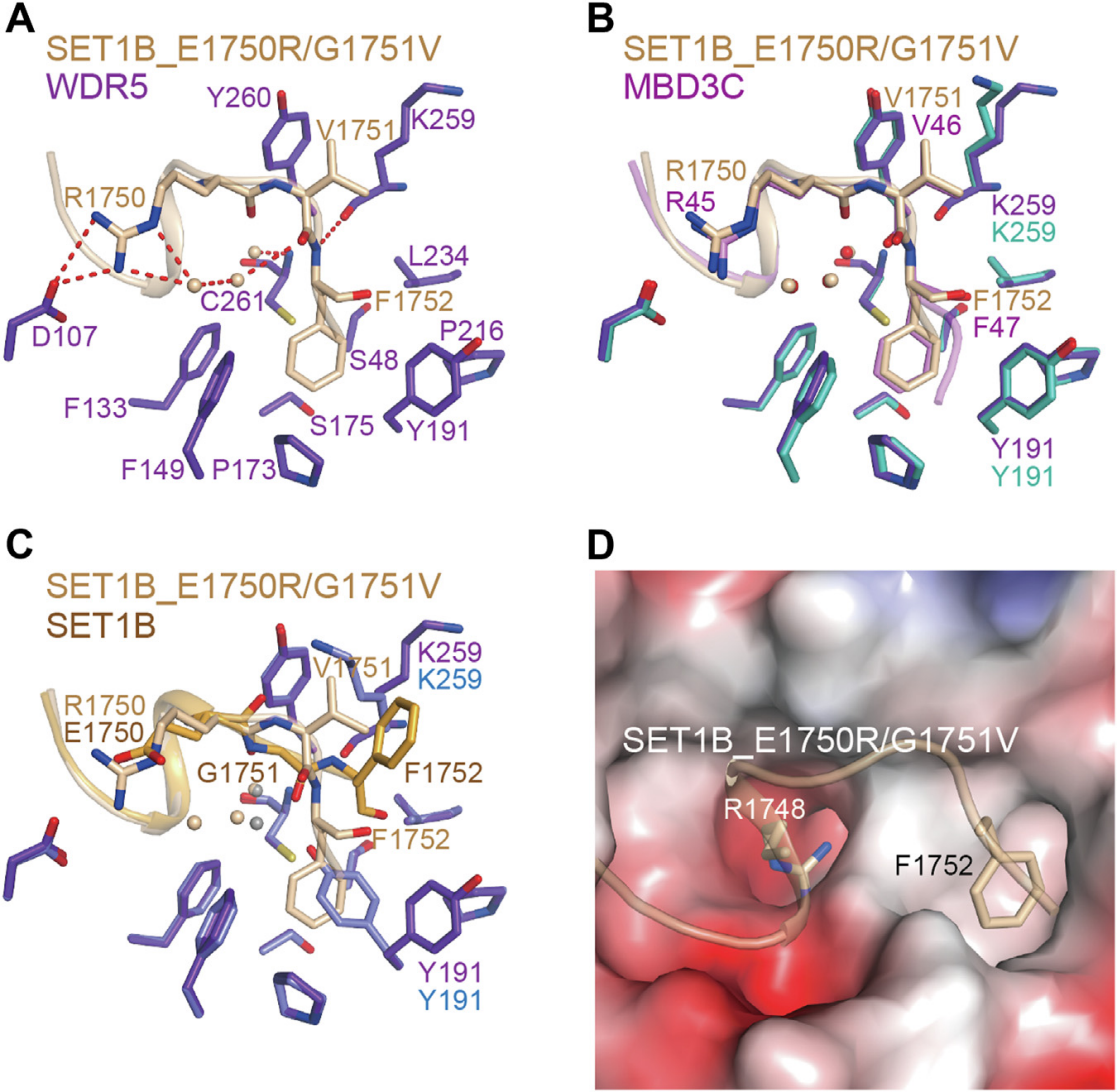
**SET1B_E1750R/G1751V peptide binds WDR5 in a way similar to MBD3C.***A*, Interaction details of the binding surface between WDR5 and SET1B- E1750R/G1751V variant peptide. WDR5 and SET1B_E1750R/G1751V are colored in *purple-blue* and *wheat*, respectively. *B*, superposition of WDR5-MBD3C and WDR5-SET1B_E1750R/G1751V. The RMSD is 0.208 Å among 296 Cα atoms. *C*, Superposition of WDR5-SET1B and WDR5-SET1B_E1750R/G1751V. The RMSD is 0.146 Å among 273 Cα atoms. *D*, electrostatic surface representation of the SET1B_E1750R/G1751V bound surface of WDR5. Arg1748 and Phe1752 of SET1B_E1750R/G1751V are labeled with *sticks*.

The structure of the WDR5-SET1B_1745-1754_ (E1750R/G1751V) complex was successfully solved at 2.10 Å resolution (Table 1). Structural analysis revealed that Arg1750 in the SET1B_1745-1754_ (E1750R/G1751V) peptide forms salt bridges with WDR5 Asp107 (Fig. 5*A*). Van der Waals contacts are observed between the side chain of Val1751 in the peptide and the aliphatic portions of WDR5 Lys259 and Tyr260. The backbone amide group of Phe1752 in the SET1B_1745-1754_ (E1750R/G1751V) peptide forms a hydrogen bond with the carbonyl oxygen of WDR5 Lys259. In addition, the side chain of Phe1752 points into the WDR5 Tyr191-related B site. Furthermore, a hydrogen-bonding network is employed by three water molecules to enhance the interactions between WDR5 and the SET1B_1745-1754_ (E1750R/G1751V) peptide. Moreover, the sulfhydryl group of Cys261 forms an S-π interaction with the aromatic ring of Phe1752 (48).

Structural alignment reveals that the binding properties between WDR5 and the SET1B_1745-1754_ (E1750R/G1751V) peptide closely resemble those of WDR5 and MBD3C_40-51_, with three water molecules occupying the vicinity of WDR5 Cys261 (Fig. 5, *B* and *C*). Consequently, it is evident that the WDR5 WIN site and B site could be recognized simultaneously by WIN motif peptides containing the ′′Arg-Cys/Ser-Arg-Val-Phe′′ sequence (Fig. 5*D*).

### MBD3C binds WDR5 in a manner analogous to the inhibitor compound C6

**Figure 6:**
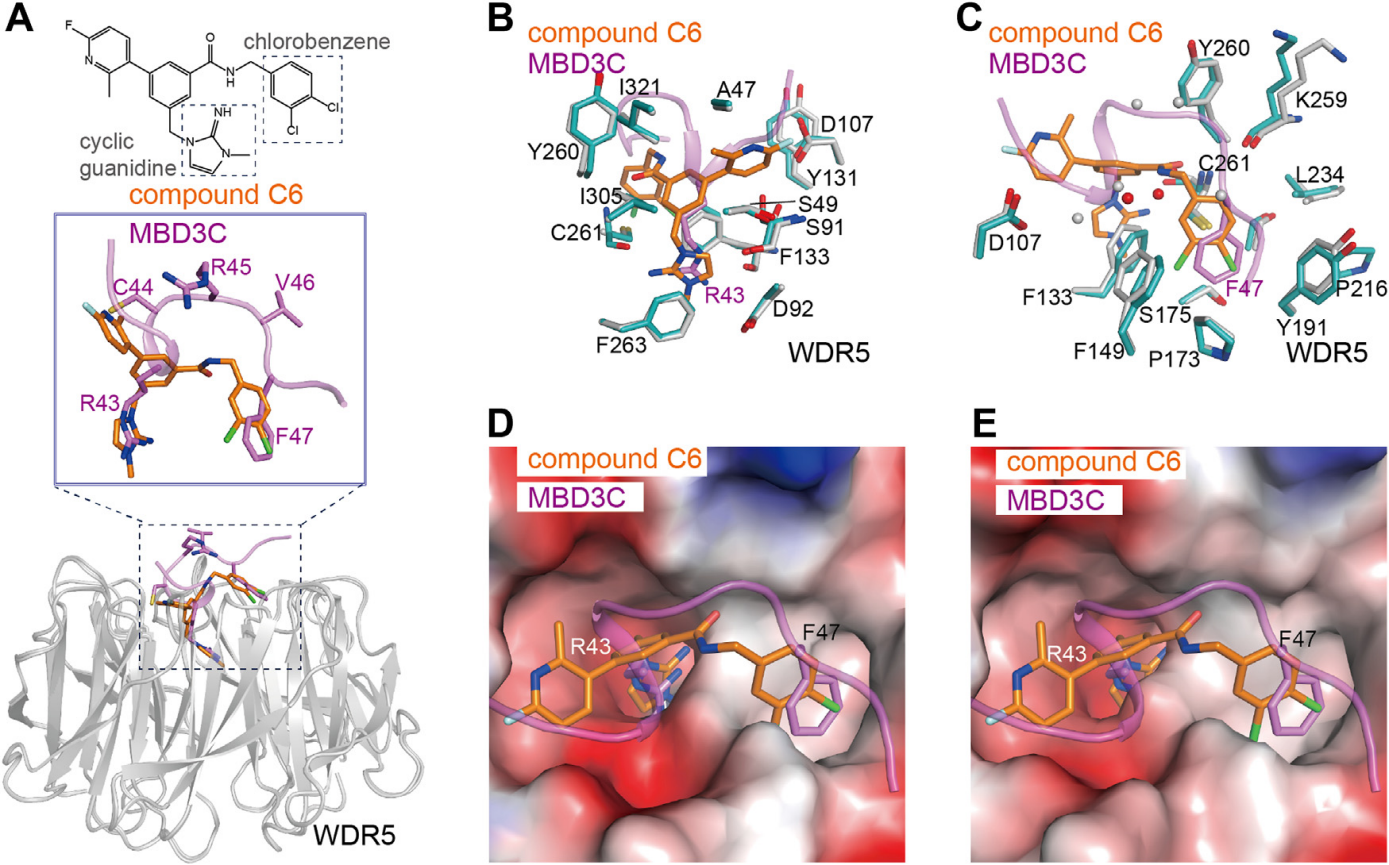
**Compound C6 binds WDR5 in a way similar to MBD3C.***A*, structural superposition of the WDR5-C6 and WDR5-MBD3C complexes. The RMSD is 0.205 Å among 270 Cα atoms. MBD3C and compound C6 are colored in violet and *orange*, respectively. Both WDR5 in WDR5-C6 and WDR5-MBD3C complexes are colored in *gray*. *B* and *C*, superposition of WDR5-C6 and WDR5-MBD3C in a view same to Figure 2*B* (*B*) and Figure 2*C* (*C*). MBD3C and compound C6 bound WDR5 are colored in *cyan* and *gray*, respectively. *D* and *E*, superposition of WDR5-C6 and WDR5-MBD3C on the electrostatic surface of MBD3C bound (*D*) and Compound C6 bound WDR5 (*E*).

The acidic pocket on the smaller surface of WDR5 has been identified as a therapeutic target for drug screening and optimization (26, 27). Recently, it was reported that obvious affinity improvement could be achieved by occupying the WDR5 B site as compound C6 does (PDB code: 6E23) (Fig. 6*A*) (50). Despite the amount of small molecule inhibitors were designed to block WDR5 WIN site, to the best of our knowledge, compound C6 was the first small molecule fragment reported by the Tansey group (50), that can occupy both WIN site and Tyr191 related B-site. Notably, the binding affinity of compound C6 bound to WDR5 is 10^6^ times higher than compound C4, which only occupies the WIN site. Furthermore, they demonstrated that compound C6 could effectively displace WDR5 from chromatin, leading to significant inhibition of leukemia cell line proliferation and induction of p53-dependent cell death.

Structure superposition is performed to clarify the correlation between the WDR5 WIN site and the B site. Compound C6 was observed to bind WDR5 in a manner similar to MBD3C (Fig. 6, *B* and *C*). Specifically, in the WDR5-C6 complex, the basic cyclic guanidine mimics MBD3C Arg43, the chlorobenzene group mimics MBD3C Phe47, and the amide connecting the basic cyclic guanidine and chlorobenzene group mimics MBD3C Cys44-Arg45-Val46. Furthermore, electrostatic surface alignment analysis revealed that the bound compound C6 exhibits a narrower acidic pocket and a wider hydrophobic pocket associated with Tyr191 (Fig. 6, *D* and *E*). The observed variation is probably attributable to the presence of chlorine in the chlorobenzene group, which introduces steric hindrance (C−Cl bond length of 1.7 Å compared to C − H bond length of 1.1 Å) at the WDR5 B site, subsequently causing conformational alterations in WDR5 Phe133 and Phe149.

In conclusion, it is likely that the WDR5 WIN site and Tyr191-related B site play a significant role in both WDR5-peptide and WDR5-compound interactions.

## Discussion

In this study, we demonstrated a direct interaction between WDR5 and the WIN motif-containing MBD3C isoform and determined the crystal structure of the WDR5-MBD3C complex. The structure provides a detailed atomic-level description of the interaction that facilitates the association between MBD3C and WDR5. The canonical consensus sequences for the WIN motif consist of a four-amino acid polypeptide with an A-R (position 0)-[A/C/S/T]-[E/K/R] (7). Previous crystal structures of WDR5 in complex with the WIN motif have underscored the essential role of arginine and alanine residues at positions 0 and −1 (2, 42, 43, 44, 45, 46, 49, 51). However, the residues flanking the conserved residues displayed variation and are the primary determinants affecting the binding affinities of WDR5 towards H3, MLL1-4, SET1A/B, and KANSL1 (3, 9, 44).

Structural analysis of the WDR5-MBD3C complex revealed a previously unknown role for Tyr191 of WDR5, which is important for the recognition of Phe47 of MBD3C at position +4 (Fig. 3*E*). Significantly, in the MBD3C-bound WDR5 complex, Tyr191 undergoes a rotation away from Cys261, generating a hydrophobic B site composed of residues surrounding WDR5 Cys261. This structural feature was not observed in the previously resolved WDR5-peptide structures. In contrast, in both the apo- and SET1B-bound forms of WDR5, Tyr191 undergoes a rotation towards Cys261, resulting in the closure of the pocket formed by the residues surrounding WDR5 Cys261. We further confirmed that Phe47 is important for WDR5 binding through the substitution of phenylalanine with alanine or histidine.

Sequence alignment revealed that SET1B contains phenylalanine at position +4 of the WIN motif (43, 44). Comparative analysis of the residues between the conserved arginine and phenylalanine at positions +1 ∼ +3 suggests that these residues may contribute to the observed structural difference. As a result, we performed residue substitution experiments and crystallization to elucidate the molecular mechanism underlying the observed structural difference. Indeed, consistent with our prediction, the structures confirmed that the residues at positions +1 ∼ +3 play important roles in the distinct conformation of phenylalanine inserted into the WDR5 B pocket.

WDR5 has been linked to a range of cancer types and is currently considered a promising target for anti-cancer therapeutics (26, 29). Current research has predominantly focused on developing WDR5 inhibitors that specifically target the WIN site on WDR5 (10, 27, 28, 50, 52), aiming to disrupt the histone methyltransferase (HMT) function of the KMT2A complex. The catalytic activity of the KMT2A complex relies on the insertion of a WIN motif into the WIN site of WDR5. The resolved WDR5-MBD3C structure has enabled us to uncover the potential of phenylalanine in the WIN motif to create a previously unobserved deep pocket site B, a feature not previously documented in other WDR5-WIN motif-containing structures. This discovery may present a promising opportunity for the development of small molecule inhibitors targeting this new binding site. For instance, the small molecule compound C6 has demonstrated strong binding to both the WIN site and WDR5 Tyr191-related B site (50), resulting in enhanced anti-cancer efficacy by promoting p53-dependent apoptosis in leukemia cells. In addition, the successful drug discovery process for the KRAS (G12C) inhibitor AMG510 is another remarkable achievement. Structural analysis led to the identification of a novel binding pocket on the surface of KRAS (G12C), which significantly contributed to the advancement of this inhibitor. The novel binding site of KRAS (G12C) is formed through an alternative orientation of KRAS (G12C) His95, enabling the binding of an aromatic ring from the AMG510 inhibitor. This alternative binding pocket substantially strengthens the interactions between the AMG510 inhibitor and the KRAS (G12C) protein (53, 54).

In summary, our structural investigation in this study has shed light on the molecular mechanism underlying the formation of the WDR5-MBD3C/NuRD complex. The subsequent ITC assays have revealed the crucial role of MBD3C Arg43 in WDR5 recognition, while Phe47 enhances the binding affinity. Consistent with the findings of Ly Sha Ee *et al.* (2017) (40), neither the MBD3C △41 to 50 mutant nor the MBD3C (R43A) was able to co-immunoprecipitate with WDR5. Therefore, our structural study underscores the significance of gene regulation through the formation of the WDR5-MBD3C complex (11, 40). In addition, our crystal structures could provide valuable insights for the advancement of potential small molecule drugs with increased binding affinity, which may have the ability to disrupt the interaction between WDR5 and WIN motif-containing proteins or strengthen the interaction with the degrader.

## Experimental procedures

### Protein expression and purification

The DNA fragment encoding the mouse WDR5 seven tandem WD-40 repeats (residues 24–334) was amplified *via* PCR from the human brain and bone marrow cDNA library and cloned into the pGEX-4T1 vector modified with an N-terminal GST tag and a TEV cleavage site (ENLYFQ/GSHM) (where ‘/’ indicates the cleavage site). All WDR5 mutants were generated using a MutanBEST kit and subsequently confirmed through DNA sequencing. The proteins were expressed in *Escherichia coli* BL21 (DE3) cells (Novagen) that were cultured in Luria–Bertani medium at 37 °C. The recombinant proteins were induced by 0.2 mM isopropyl β-D-1-thiogalactopyranoside (IPTG) upon reaching an optical density at 600 nm (OD600 nm) 0.8, then transferred to 16 °C and incubated for 18 h. Cells were harvested by centrifugation and resuspended in Buffer A (20 mM Tris-HCl, 500 mM NaCl, pH 8.0), supplemented with DNAaseI (10U) and protein protease inhibitor (Roche, 1 tablet dissolved in 1 ml sterile water), and then lysed by a high-pressure cell homogenizer. The cell debris was removed by centrifugation, and the supernatant was subjected to a 6-h incubation at 4 °C with glutathione Sepharose resins (GE Healthcare) The resins bound by GST-tagged proteins were resuspended in 20 ml Buffer A. Then, the GST tag was removed through digestion with ∼1 mg TEV protease (purified in-house) at 4 °C overnight. The cleaved proteins were concentrated using a 10-kD Amicon Ultra concentrator (Millipore) and then purified using size-exclusion chromatography on a HiLoad 16/60 Superdex S200 column (GE Healthcare) in Buffer A. Additionally, purification was carried out using a MonoS 5/50 Gl column (GE Healthcare) in low-salt Buffer B (20 mM Tris-HCl, 100 mM NaCl, pH 7.0) and high-salt Buffer C (20 mM Tris-HCl, 1 M NaCl, pH 7.0). The peak was collected and dialyzed in Buffer D (20 mM Tris-HCl, 200 mM NaCl, pH 7.5), followed by concentration for subsequent crystallization or ITC experiments.

### Peptide synthesis

The peptides used in the study were synthesized by GenScript Biotechnology (Nanjing) Co, Ltd. The lyophilized synthesized peptides (purity>95%, verified by mass spectrometry) were carefully weighed in a large amount and dissolved in Buffer D. The pH of each sample was adjusted to a value of 7.5 with NaOH. The sequences of the peptides are as follows:

MBD3C_40-51_: GAARCRVFSPQG

MBD3C_40-51_ (R43A): GAAACRVFSPQG

MBD3C_40-51_ (R43D): GAADCRVFSPQG

MBD3C_40-51_ (R43E): GAAECRVFSPQG

MBD3C_40-51_ (R43K): GAAKCRVFSPQG

MBD3C_40-51_ (R45E): GAARCEVFSPQG

MBD3C_40-51_ (F47A): GAARCRVASPQG

MBD3C_40-51_ (F47H): GAARCRVHSPQG

MBD3C_40-51_ (R45E/V46G): GAARCEGFSPQG

MBD3C_40-51_ (C44S/R45E/V46G): GAARSEGFSPQG

SET1B_1745-1754_: GCARSEGFYT

SET1B_1745-1754_ (E1750R/G1751V): GCARSRVFYT

### Protein crystallization, data collection and structure determination

The WDR5 protein was concentrated to 16 to 20 mg/ml. Prior to crystallization, the protein-peptide complexes were prepared by mixing the protein with peptides at a molar ratio of 1:3 and incubated on ice for 30 min. The precipitate was removed by centrifugation before crystallization. The crystals were grown at 293 K *via* the sitting drop method by mixing 1 μl of protein-peptide complex and 1 μl of reservoir buffer. WDR5-MBD3C_40-51_ complex crystals were grown at 0.1 M HEPES (pH 7.5) and 18% PEG 600. WDR5-MBD3C_40-51_ (F47A) complex crystals were grown at 0.2 M potassium dihydrogen phosphate, 20% PEG 3350. WDR5-MBD3C_40-51_ (R45E/V46G) complex crystals were grown at 0.2 M imidazole malate, pH 5.5, 33% PEG 600. WDR5-MBD3C_40-51_ (C44S/R45E/V46G) complex crystals were grown at 0.2 M lithium chloride, 20% PEG 3350. WDR5-SET1B_1740-1754_ complex crystals were grown at 0.1 M Bis-Tris, pH 5.5, 25% PEG 3350. WDR5-SET1B_1740-1754_ (E1750R/G1751V) complex crystals were grown at 0.1 M sodium citrate, pH 5.5, 16% PEG 4000, 10% 2-propanol. The crystals were soaked in cryo-protector made of mother liquor supplemented with 25% (v/v) glycerol before being flash-frozen in liquid nitrogen.

All the X-ray diffraction data of the aforementioned complexes were collected on beamline BL18U1/19U1 at the Shanghai Synchrotron Radiation Facility (SSRF). The diffraction data set was processed and scaled using the *HKL*-2000 program suite (55). The structure of WDR5 was determined by molecular replacement using monomeric WDR5 (PDB code: 2H14) as a search model with the Phase program (56). Next, the peptides were built manually by Coot and then refined with Refmac5 in the CCP4 package (57, 58). Final refinement was performed using the Phenix.refine program (59). All the structures in the figures were generated using the PyMOL program (DeLano Scientific LLC). The statistics for data collection and structural refinement of six crystal structures are summarized in Table 1.

### Isothermal titration calorimetry

ITC assays were performed on a Microcal PEAQ-ITC instrument (Malvern Panalytical) at 20 °C. Proteins were quantified by absorbance at 280 nm and diluted to 44.5 μM with Buffer D. The peptides were adjusted to 0.5 to 1.0 mM with the same buffer. The titration protocol was the same for all the measurements, which consisted of a single initial injection of 1 μl peptide, followed by 19 injections of 2 μl peptide into protein samples. The reference power was 5 μcal/s, and the intervals between injections were set to 150 s. Curves fitting to a single binding site model were performed by MicroCal PEAQ-ITC Analysis Software provided by the manufacturer and summarized in Table 2.

### Circular dichroism spectroscopy

The far-UV spectra of WDR5 and its mutants were measured using a MOS-500 spectrometer at 20 °C. The spectra were recorded from 200 nm to 260 nm at a 2 nm bandwidth using a 1-cm path length cell. The acquisition period is 0.5 s. WDR5 and its mutants were prepared at a concentration of 1 μM in 20 mM sodium phosphate buffer (pH 7.4) containing 100 mM NaCl and then degassed by centrifugation. A buffer-only sample served as a reference, and all the samples were tested in triplicate. The datasets were processed using Originlab software.

## Data availability

The atomic coordinates and structure factors for the WDR5-MBD3C_40-51_, WDR5-MBD3C_40-51_ (F47A), WDR5-MBD3C_40-51_ (R45E/V46G), WDR5-MBD3C_40-51_ (C44S/R45E/V46G), WDR5-SET1B_1745-1754_, and WDR5-SET1B_1745-1754_ (E1750R/G1751V) complexes have been deposited in the Protein Data Bank with the accession numbers 8WXQ, 8WXR, 8WXT, 8WXU, 8WXV, 8WXX, respectively.

## Supporting information

This article contains supporting information.

## Conflict of interest

The authors declare that they have no known competing financial interests or personal relationships that could have appeared to influence the work reported in this paper.
