# Supplementary material for: Structural studies of WDR5 in complex with MBD3C WIN motif reveal a unique binding mode
Source: J Biol Chem. 2024 Jun 12;300(7):107468. doi: 10.1016/j.jbc.2024.107468 (PMC11261779; doi:10.1016/j.jbc.2024.107468)
Supplement: Supporting information [file mmc1.docx]

**Supporting Information**

**Structural studies of WDR5 in complex with MBD3C WIN motif reveal a unique binding mode**

Yang Yang^1,#,*^, Li Xu^2,#,*^, Shuting Zhang^1^, Liangrui Yao^1^, Yuqing Ding^1^, Wenwen Li^1^, Xuemin Chen^1,*^

^1^ School of Life Sciences, Anhui University, Hefei, Anhui, 230601, China

^2^ Institute of Biotechnology and Health, Beijing Academy of Science and Technology, Beijing 100089, China

^#^ These authors contributed equally to this work.

**Correspondence**: Yang Yang, ahuyy@ahu.edu.cn; Li Xu, xl199207@mail.ustc.edu.cn; Xuemin Chen, cxmsherman@163.com


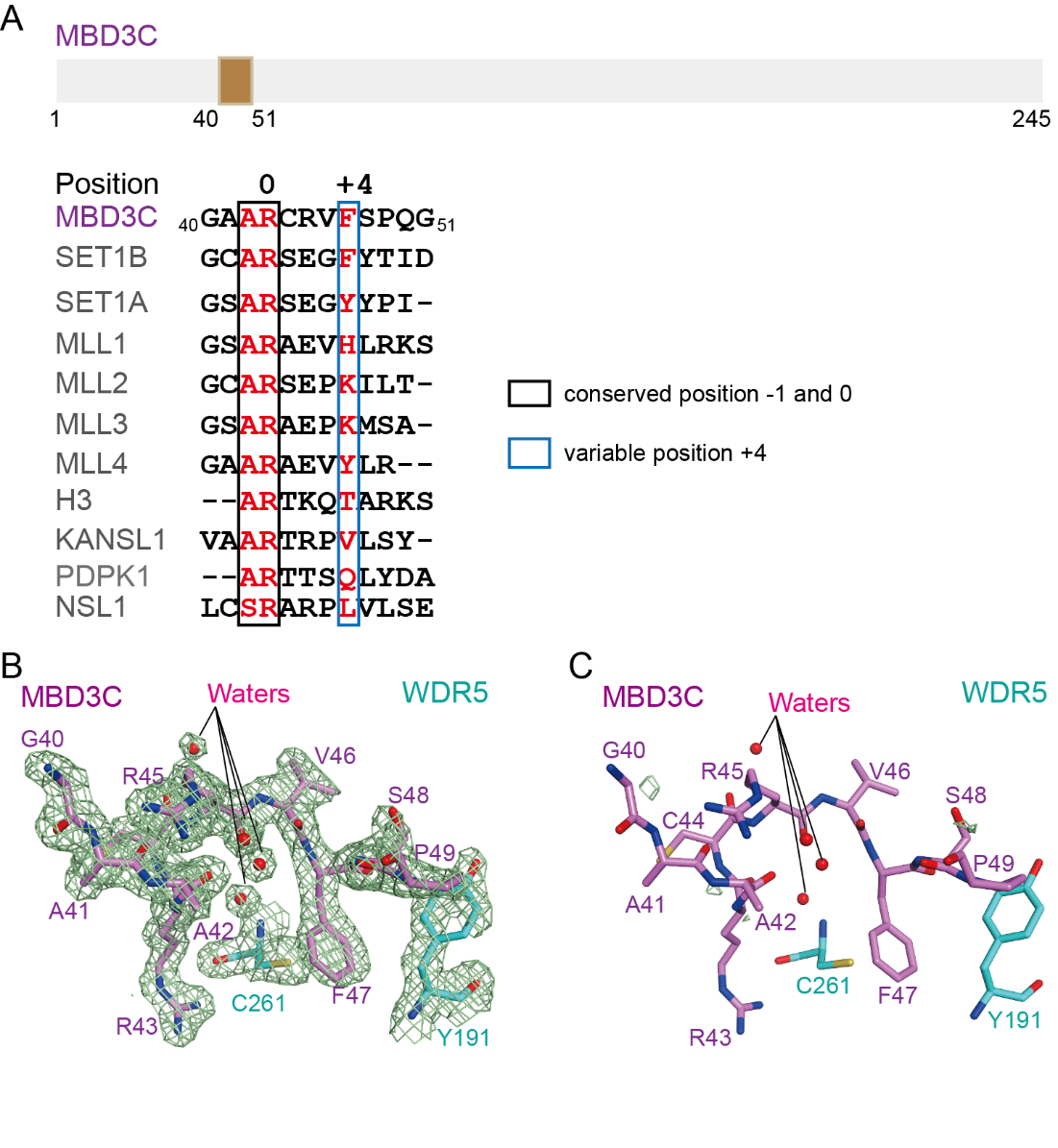


**Figure S1.** **Schematic representation of the WIN motif of MBD3C and electron density map of MBD3C peptide.**

(A) The sequence of MBD3C WIN motif and sequence alignment of WIN motif-containing peptides are shown. Position -1, 0 and +4 of WIN motif-containing peptides are labeled with gray box and light-blue box, respectively. (B) The 2Fo-Fc omit map of MBD3C peptide, waters and WDR5 Cys261, Tyr191 are contoured at 1.0 σ level. (C) The Fo-Fc omit map of MBD3C peptide, waters and WDR5 Cys261, Tyr191 are contoured at 3.0 σ level.


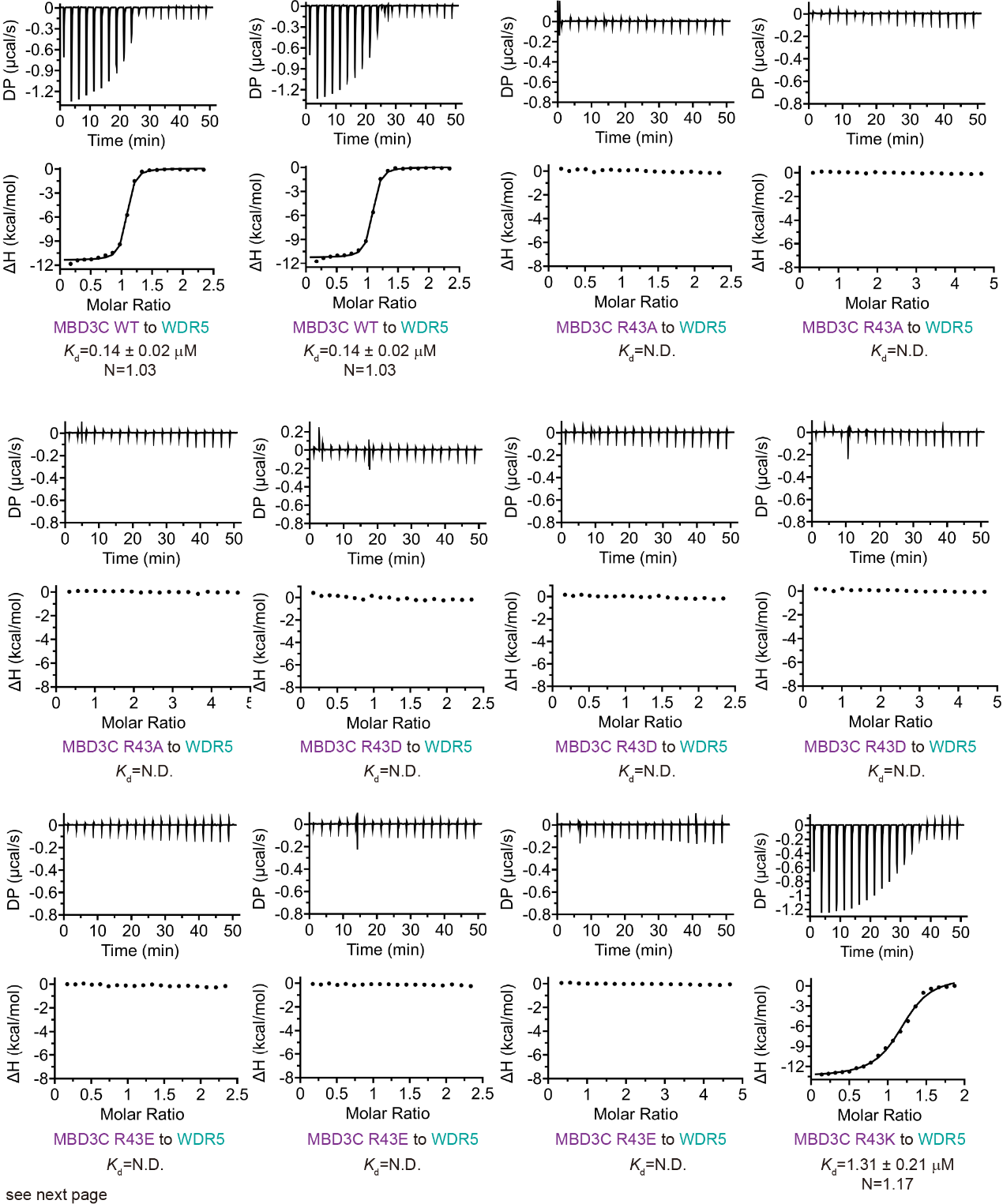


**To be continued**


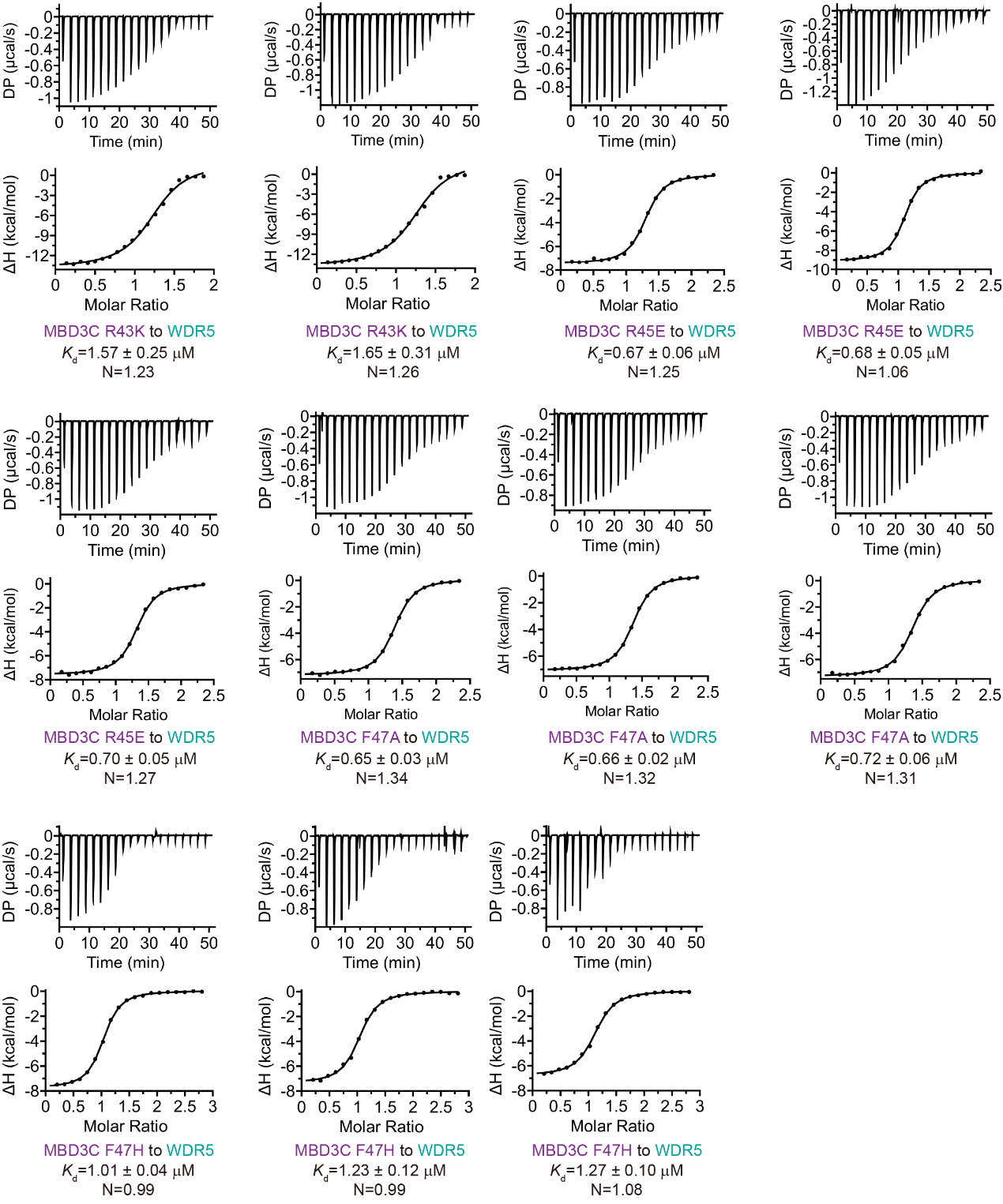


**Figure S2. ITC measurement of the interactions between WDR5 and MBD3C mutants.**

**
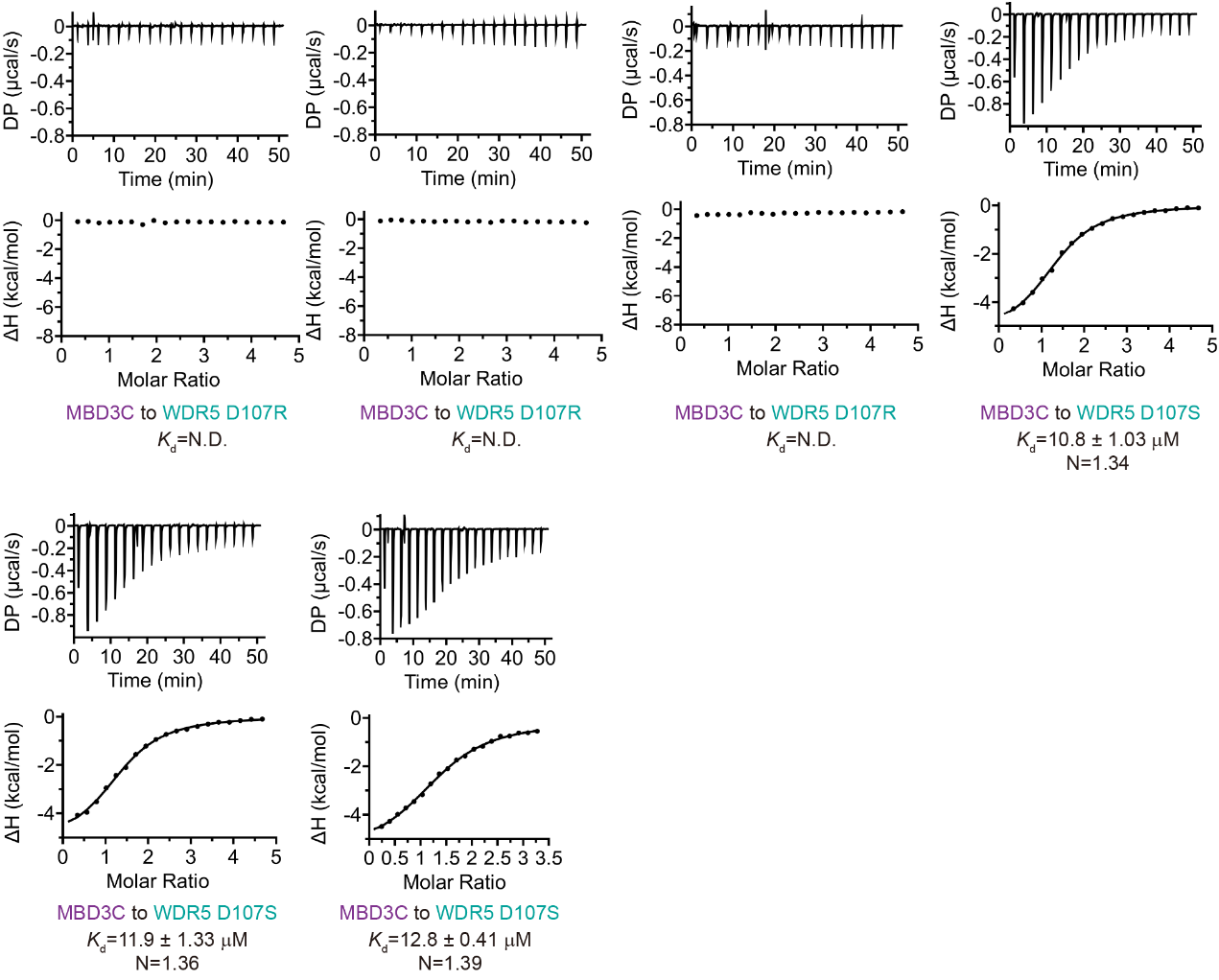
**

**Figure S3.** **ITC measurement of the interactions between WDR5 mutants and MBD3C peptides.**


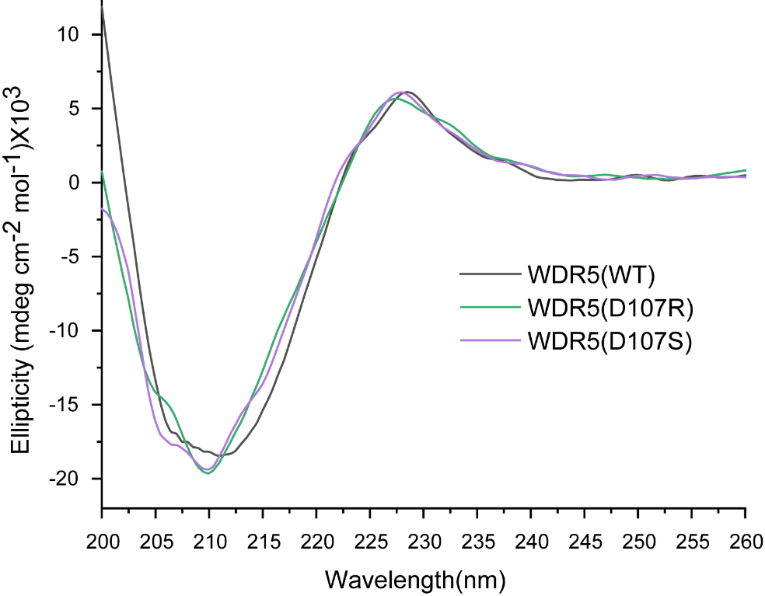


**Figure S4**. **Circular dichroism spectra of wild-type and mutant WDR5 constructs at 1 μM.**


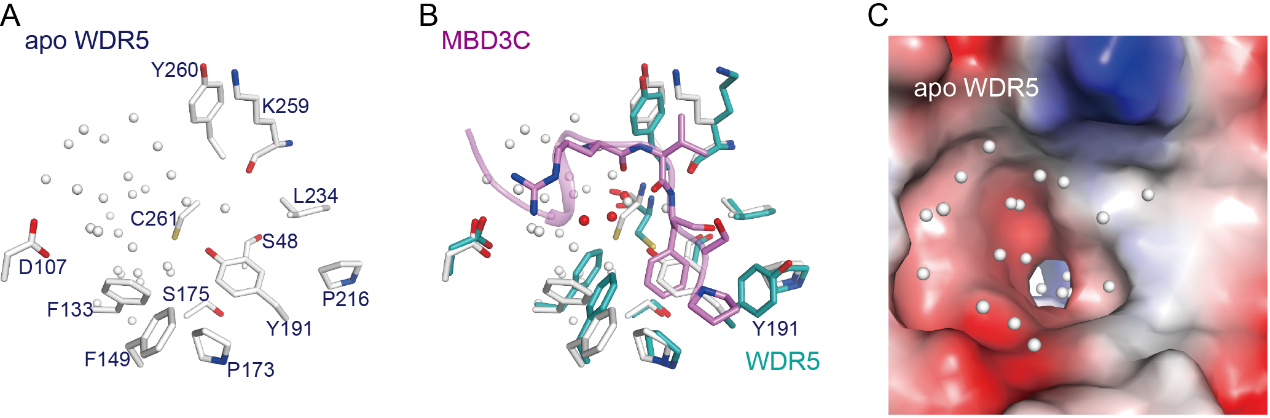


**Figure S5**. **Structural difference between apo and MBD3C bound WDR5.** (A) Details of Tyr191 area of apo WDR5 (PDB code: 2H14) in a view same to Fig. 2C. WDR5 is colored in white, waters are shown as white spheres. (B) Superposition of apo and MBD3C bound WDR5. Waters from WDR5-MBD3C complex are shown as red spheres. The RMSD is 0.457 Å among 272 Cα atoms. (C) Electrostatic surface representation of the apo WDR5 in a view same to Fig. 1F. It is clear that WIN site is filled with waters in the apo WDR5.


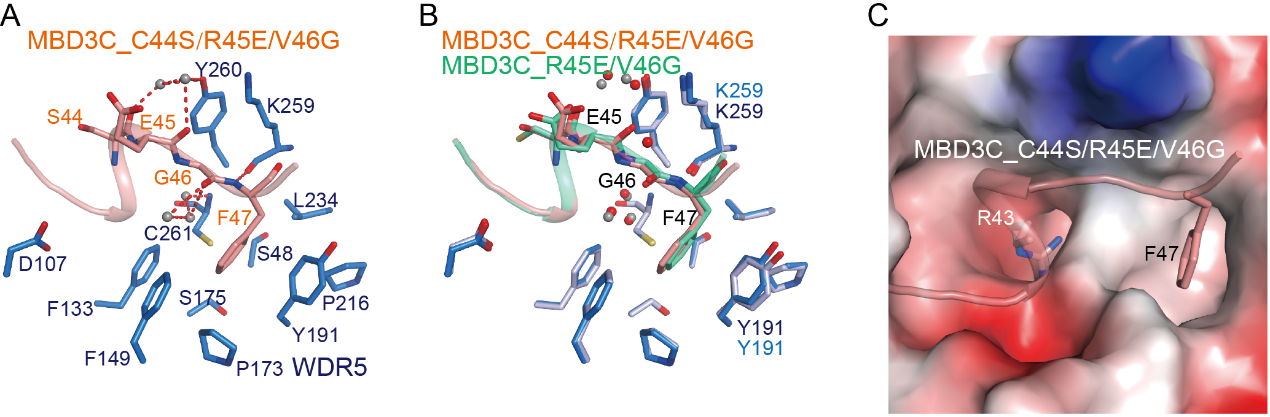


**Figure S6**. **Interaction details between WDR5 and MBD3C_C44S/R45E/V46G.** (A) Details of the binding surface between WDR5 and MBD3C_C44S/R45E/V46G variant peptide. WDR5 and MBD3C_C44S/R45E/V46G are colored in marine and salmon, respectively. (B) Superposition of WDR5-MBD3C_R45E/V46G and WDR5-MBD3C_ C44S/R45E/V46G. The RMSD is 0.135 Å among 296 Cα atoms. (C) Electrostatic surface representation of the MBD3C_C44S/R45E/V46G bound surface of WDR5. Arg43 and Phe47 of MBD3C_C44S/R45E/V46G are shown with stick.
